# Advertising patient and public involvement in trials as a way of improving participant recruitment: development of an intervention and its evaluation

**DOI:** 10.1186/1745-6215-16-S2-P100

**Published:** 2015-11-16

**Authors:** Adwoa Hughes-Morley, Bridget Young, Mark Hann, Waquas Waheed

**Affiliations:** 1 MRC North West Hub for Trials Methodology Research, Manchester Academic Health Science Centre, University of Manchester, Manchester, UK; 2 MRC North West Hub for Trials Methodology Research, Institute of Psychology, Health and Society, University of Liverpool, Liverpool, UK; 3 NIHR School for Primary Care Research, Manchester Academic Health Science Centre, University of Manchester, Manchester, UK; 4 Lancashire Care NHS Foundation Trust, Preston, UK

## Background

Evidence is emerging that patient and public involvement (PPI) may improve recruitment into trials, but the mechanism of action is unclear. Where trials use PPI to improve design and conduct, many do not communicate that clearly to potential participants. Better advertising of PPI in trials might encourage patient participation.

## Aims

We aim to develop an intervention advertising PPI in mental health trials to potential participants, and evaluate its impact on recruitment by embedding trials of the intervention in ongoing mental health host trials.

## Methods

The principles underlying the intervention were informed by a systematic review and a workshop that included mental health service users, trialists and ethicists. We recruited mental health trials with high quality PPI, and worked with PPI members in each trial to develop the intervention using a leaflet to advertise the nature and function of the PPI. Professional graphic design optimised readability and impact. Potential participants in each host trial will be randomised to receive the leaflet or not, alongside the standard study information. The effectiveness of the leaflet will be evaluated within and across trials.

## Results

Three host trials have been recruited: leaflets have been successfully developed in two, with the third currently in development. To date 1871 patients have been randomised.

## Discussion

By the time of the conference, we will present data on effectiveness, and discuss the different ways in which PPI can enhance recruitment in trials. The intervention will improve the evidence base for recruitment, as well as the impact of PPI.

